# The C2DREAM framework: Investigating the structural mechanisms undergirding racial health inequities

**DOI:** 10.1017/cts.2024.518

**Published:** 2024-04-16

**Authors:** Kene Orakwue, Anna K. Hing, Tongtan Chantarat, Derek Hersch, Ebiere Okah, Michele Allen, Christi A. Patten, Felicity T. Enders, Rachel Hardeman, Sean M. Phelan

**Affiliations:** 1 Division of Health Policy and Management, University of Minnesota, Minneapolis, MN, USA; 2 Center for Antiracism Research for Health Equity, Minneapolis, MN, USA; 3 Department of Family Medicine and Community Health, University of Minnesota Medical School, Minneapolis, MN, USA; 4 University of Minnesota Clinical Translational Science Institute, Minneapolis, MN, USA; 5 Department of Psychiatry & Psychology, Mayo Clinic, Rochester, MN, USA; 6 Department of Quantitative Health Sciences, Mayo Clinic, Rochester, MN, USA; 7 Robert D. and Patricia E. Kern Center for the Science of Health Care Delivery & Division of Health Care Delivery Research, Mayo Clinic, Rochester, MN, USA

**Keywords:** Clinical translational research, cardiovascular disease, hypertension, obesity, racism, social determinants of health

## Abstract

Racism shapes the distribution of the social determinants of health (SDoH) along racial lines. Racism determines the environments in which people live, the quality of housing, and access to healthcare. Extensive research shows racism in its various forms negatively impacts health status, yet few studies and interventions seriously interrogate the role of racism in impacting health. The C2DREAM framework illuminates how exposure to racism, in multiple forms, connects to cardiovascular disease, hypertension, and obesity. The goal of the C2DREAM framework is to guide researchers to critically think about and measure the role of racism across its many levels of influence to better elucidate the ways it contributes to persistent health inequities. The conceptual framework highlights the interconnectedness between forms of racism, SDoH, and the lifecourse to provide a greater context to individual health outcomes. Utilizing this framework and critically contending with the effects of racism in its multiple and cumulative forms will lead to better research and interventions.

## Introduction

People racialized as Black in the United States (US) are at increased risk for cardiovascular disease (CVD) and CVD risk factors, including hypertension, and obesity and are twice as likely to die from CVD compared to people racialized as white [1,2]. CVD is the leading cause of death for those who identify as Indigenous and Black [3]. These inequities have persisted over time, despite medical and behavioral intervention [2]. Thus, researchers and interventionists must consider how upstream forces such as racism influence the distribution of social determinants of health (SDoH) and CVD. The foundational underlying risk factor to health inequities is chronic exposure to racism, not race [4].

Racism is grounded in and upholds white supremacy – “the glossary of conditions, practices and ideologies that underscore the hegemony of whiteness and White political, social, cultural, and economic domination [5].” Structural racism – the “totality of ways in which societies foster racial discrimination through mutually reinforcing systems of housing, education, employment, earnings, benefits, credit, media, health care, and criminal justice [6],” is woven into every facet of US society, and normalized, even invisible, to privileged groups [7]. It is multidimensional, dynamic, and persistent, manifests across sectors, evolves over time, and resists attempts to dismantle it. Structural racism, present and historical, shapes the distribution of SDoH.

In addition to structural racism, there are four other forms of racism – cultural, institutional, interpersonal, and internalized. These forms are mutually reinforcing and shape the opportunities, barriers, stressors, and health of Americans, rewarding those who are racialized as white while disadvantaging those who are not. To dismantle racism at its roots and interrupt the mechanisms leading to its harmful impacts on health, researchers, practitioners, and health equity professionals need a greater understanding of how racism manifests and how the different forms interact. Yet little research has examined the interactive and cumulative effects of different forms of racism on health.

Research and interventions centered on exposure to racism, rather than race, are urgently needed. Race is not defined by genetics; rather, race is a social construction of identity and group association categories loosely based on phenotypes, shared culture/history, and ancestry [8]. Racism is directly connected to SDoH; as the distribution of social determinants is patterned along racial lines due to the founding origins of the USA. Extensive research shows racism in its various forms negatively impacts health status, yet few studies and interventions seriously interrogate the role of racism in impacting health [9]. The recent emergence of models that address racism and SDoH related to health inequities, including those across the lifecourse [10], and approaches to interrogating research processes through an anti-racist lens, such as Public Health Critical Race Praxis [8], represent important developments in the fields. However, additional frameworks, along with potential measures, are needed to guide broad conceptualization of the relationships between racism, SDoH, and healthcare delivery across multiple outcomes.

Failure to address the role of racism in perpetuating inequities upholds and reinforces white supremacy. There is a need to understand the full manifestation of racism and health, to dismantle oppressive systems, and to implement equity-promoting interventions.

## The Center for Chronic Disease Reduction and Equity Promotion Across Minnesota

The Center for Chronic Disease Reduction and Equity Promotion Across Minnesota (C2DREAM) is a multidisciplinary and multi-institutional center funded by the National Institute of Minority Health and Health Disparities (NIMHD), founded to encourage conceptual alignment of health equity research conducted in and focused on the population of Minnesota and across the USA. Its membership consists of community members, faculty, and staff from multiple healthcare and community organizations across rural and urban areas of Minnesota, including the University of Minnesota, Mayo Clinic, Hennepin Healthcare, and the Native American Community Clinic.

The mission of C2DREAM is to eliminate the inequitable burden of CVD, hypertension, and obesity experienced by communities racialized as Black, Indigenous, and People of Color (BIPOC), including immigrants and refugees across Minnesota. Its overarching aim is to generate knowledge, analytical and implementation approaches, and community engagement strategies to develop solutions that interrupt the effect of racism on cardiovascular health. C2DREAM is achieving this aim using a common conceptual framework guiding all affiliated studies, as well as a set of common data elements. C2DREAM investigators are encouraged to consider how their aims map onto the common model and contribute data to cross-study analyses aiming to uncover and highlight relationships between multilevel factors relevant to racial health inequities in chronic disease that would be difficult to identify in any single study or sample. C2DREAM is also part of a network of 11 NIMHD-funded centers, each of which is contributing to a repository of common SDoH data, with the goal of examining contributors to racial health inequities in a broader context.

Here we present our conceptual framework that guides C2DREAM research to consider how racism operates and contributes to inequities in CVD, hypertension, and obesity. This framework was developed by C2DREAM with the input of public health researchers, health care providers, and community organization representatives from institutions across Minnesota. This framework intends to move the needle by providing a pathway to develop best practices for research on SDoH. Furthermore, this framework addresses two major limitations of current research practices. First, research frequently isolates CVD, hypertension, and other chronic diseases, leading to disease-specific interventions. Second, common practice focuses on individual behavioral factors that are insufficient to address complex social contributors to inequities. Our framework recognizes the need for a research approach that considers upstream common contributors to multiple chronic diseases and strives to outline how structural factors, especially racism, influence health. Due to the fact that racism intersects with and drives SDoH, we need to create research frameworks that focus on racism at multiple levels as the common driver of these inequities. The C2DREAM framework seeks to answer this need, by centering racism in its analyses and interventions, and modeling this practice for future research.

## Framework

### Grounding theory

**Figure 1.**
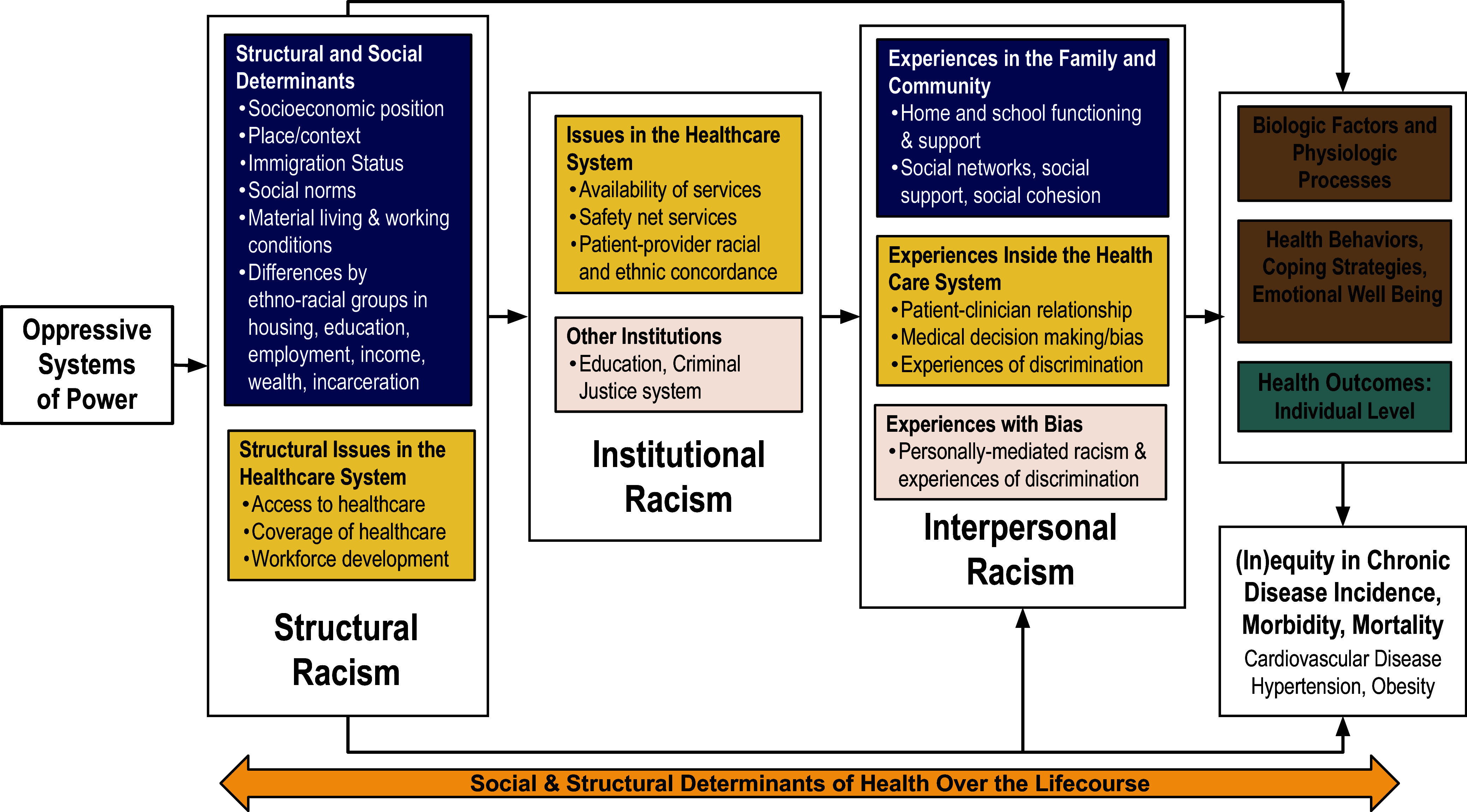
C2DREAM framework.

The C2DREAM framework (Fig. 1) illuminates how exposure to racism, in multiple forms, connects to CVD, hypertension, and obesity [2]. The C2DREAM framework begins to conceptualize the lived experiences of many racialized populations by interrogating the contribution of racism to health inequities in chronic disease incidence and outcomes – upstream from SDoH. Though C2DREAM specifically focuses on cardiovascular diseases, hypertension, and obesity, the model applies to any chronic disease for which racial disparities exist. It draws upon the social-ecological model [11], NIMHD Research framework [12], lifecourse perspective [13–15], and public health critical race praxis [8] to inform its conceptualization.

#### Social-ecological model

The social-ecological model offers a depiction of concentric circles to represent the interconnection and influence of external domains impacting health from the most proximal individual to the more distal societal levels [11]. It highlights that health is the sum of interactions across domains of life. The many renditions of the social-ecological model broadly include individual, family/interpersonal, community, and society/policy levels [11]. The eco-social model, by Dr. Nancy Kreiger, recognizes the ways in which race and racism produce health inequities across these domains over the lifecourse [16]. It emphasizes the importance of structural determinants of racial inequality (policies, laws, etc.) and their impact on determinants of health and individual health status. The C2DREAM framework relies on this conceptualization to evaluate the wide pervasiveness and multilevel influence of racism.

#### NIMHD research framework

Influenced by the social-ecological model, the NIMHD Research Framework offers a deeper understanding of how health inequities are produced. By breaking down domains of influence (biological, behavioral, physical/built environment, sociocultural environment, and healthcare system) and levels of influence (individual, interpersonal, community, and societal) over the lifecourse, the framework demonstrates pathways to health inequities by highlighting factors across health outcome populations (individual health, family/organizational health, community health, and population health) [12]. This framework offers a logical structure for understanding how inequities are produced. The C2DREAM framework reconceptualizes domains of influence in a pathway, to show how each form of racism is not independent, but influenced by multiple forms.

#### Lifecourse theory

To understand the ways in which racism impacts individuals as they age and over time, we borrow critical concepts from the lifecourse perspective [13]. Entering and leaving institutions (i.e., educational or job opportunities) determine who, where, and how people interact with society and different forms of racism present across sectors/institutions. Further, exposure to racism during sensitive or critical periods may have heightened health impact [13]. Exposure to racism earlier in life could also make one more susceptible to negative impacts from other forms of racism later in life, due to increased weathering [20]. For example, those racialized as Black have increased rates of low birth weight. Being born with a low birth weight puts one at increased risk for CVD later in life. Thus, the lifecourse perspective requires us to think about age-patterned exposures to racism, and sensitive and critical periods, which are critically important to the relationship between racism and health at all stages of life. A recent publication by Iruka *et al*. (2022) puts forth a multidimensional conceptualization of the impact of racism on racially and ethnically minoritized children’s development [10]. The *R* ^3^ISE integrative model focuses on the influences of cultural, systemic, interpersonal, and internalized racism as key mechanisms for impacting childhood development, growth, and learning. Iruka *et al.*, also highlight the effects of vicarious, every day, symbolic, cyber, and aversive racism for consideration [10]. The cumulative impact of racism is not explicitly described in the C2DREAM framework, but represented as an arrow at the bottom of Figure 1.

#### Public health critical race praxis (PHCRP)

Public health critical race praxis (PHCRP) applies the tenets of critical race theory to public health as a means to strengthen the understanding of how racism is at work, including the ordinariness of racism, race consciousness, and the primacy of racism [8]. Race (or racism) consciousness requires researchers and practitioners to consider the differential racialization process and how racism is present in an individual’s life, being explicit about that interrogation to consider how exposures to different stressors interact with racism to harm health. For example, thinking about why a patient may be living with obesity requires investigators to look beyond personal behaviors and choices, to consider how racism may have structured the built environment, hindering access to healthy foods and green spaces, or exposing the individual to high levels of chronic stress hormones that increase adiposity. Furthermore, PHCRP and, specifically, racism consciousness encourages researchers and practitioners to investigate the very term “obesity,” and unpack its fatphobic and anti-Black origins. Next, the ordinariness of racism suggests that rather than being the exception, racism is the rule [8]. Racism and white supremacy are foundational to how society is structured in the USA. Lastly, PHRCP emphasizes primacy, meaning that as a practice, we need to prioritize understanding the link between racism and health, which is the goal of this framework. While specific components of this framework may not explicitly be seen in Figure 1, the authors used PHCRP when creating the framework and hope that the concepts discussed above are reflected in the application of the framework.

In addition to the aforementioned theories, the research team drew from their expertise and conceptualizations to design the C2DREAM framework. The creation was an interactive process in which the research team discussed in depth and created multiple versions to capture the complexity of how racism influences CVD.

### Organization, definitions, and measurement

At the macro-level, the C2DREAM framework (Fig. 1) is organized by forms of racism. While most research on racism and health has historically focused on interpersonal racism, researchers have begun to move upstream to examine how SDoH and institutional, structural, and cultural racism are related to health and the development of health inequities. The C2DREAM framework considers “how [each form of racism] is at work here [17],” and to delineate the pathways through which these forms influence health. The conceptual framework is organized as follows from left to right: oppressive systems, structural racism, institutional racism, and interpersonal racism (See Table 1). This framework does not currently measure cultural or internalized racism, as these pathways connected to CVD are less established. The C2DREAM framework prioritizes structural and institutional racism.

**Table 1.** Forms of racism

Forms of Racism	Definition	Example of Chronic Health Consequences and Impact on SDoH
Oppressive Systems of Power	Other oppressive systems and supporting ideologies, including but are not limited to: ideologies of white supremacy, anti-Blackness, xenophobia, homophobia, imperialism/colonialism, capitalism, patriarchy, and Christianity superiority.	Experiences of oppression can cause elevated levels of stress. Oppressive systems have resulted in giving Indigenous Americans high levels of historical trauma, specifically settler-colonialism-induced trauma. Chronic elevated stress levels increase allostatic load which is associated with increased risk for CVD, hypertension, and obesity. The disruption to traditional way of life, displacement/relocation, and intergenerational trauma passed to Indigenous communities impacts SDoH [18].
Structural Racism	The “totality of ways in which societies foster racial discrimination through mutually reinforcing systems of housing, education, employment, earnings, benefits, credit, media, health care, and criminal justice [19]”	Structural racism makes access to higher education more difficult for those racialized as non-white. Birthing people racialized as Black even with high levels of educational attainment have worse maternal health outcomes than birthing people racialized as white with lower educational attainment. Birthing people racialized as Black have higher rates of low birth weight infants as compared to those racialized as white. Low birth weight is associated with increased risk for adult CVD [20].
Institutional Racism	“refer[s] to a system of procedures, practices, and ideologies within and among organizations that disadvantage and abuse non-White groups [21]”	Institutional racism impacts the quality of experiences in institutions. People racialized as Black receive differential treatment in healthcare settings. The embedded discrimination and biases in the current medical system are responsible for the underutilization of CVD treatment by Black patients [20].
Interpersonal Racism	“directly perceived discriminatory interactions between individuals. […] In all cases, perpetrators of discrimination act unfairly toward members of socially defined subordinate groups to reinforce relations of dominance and subordination, thereby bolstering privileges conferred to them as members of a dominant group [22]”	Experiences of interpersonal racism may lead to psychological distress. The chronic exposure to these events, potentially coupled with varying coping mechanisms- such as increased vigilance are associated with poorer health outcomes. This associated stress may lead to hypertension, a risk factor of CVD [20].

CVD = cardiovascular disease; SDoH = social determinants of health.

This framework begins by considering oppressive systems of power, pictured left (Fig. 1). This box acknowledges that other oppressive ideologies and systems, not only racism, may shape people’s lives and contribute to observed health inequities. Intersectionality theory suggests that it is not the summation of these systems but the simultaneous existence of oppressive systems of power that shape social positioning and lived experience [23]. While the C2DREAM framework focuses primarily on compounding forms of racism as a mechanism underlying health inequities, it acknowledges there may be additional contributors at play.

The framework next examines structural racism. Structural racism has historic and contemporary manifestations. The historic manifestations are many and include colonization, chattel slavery, Jim Crow laws and redlining. It also includes a deep history of medical experimentation by James Marion Sims (and others), forced and coerced sterilization of primarily Black and Puerto Rican women, unethical and non-consensual experimentation in the Tuskegee Syphilis Experiment and Henrietta Lacks, and the Flexner report [19]. The legacy of structural racism, and its accompanying pervasive ideologies have and continue to impact our society, the United States healthcare system, and research initiatives.

The framework next considers institutional racism. Institutional racism and structural racism are often conflated [24], however institutional racism refers to specific organizational policies and environments within organizations (i.e. a hospital or a school) as opposed to the broader societal policies that govern systems within one domain (i.e. the medical system or education system) [24]. The delineation of structural racism versus institutional racism is a key difference between the C2DREAM framework and the *R* ^3^ISE integrative model [10].

In healthcare, institutional racism can be seen in how policies and practices within an institution differentially impact racial and ethnic groups [21]. Institutional racism is observed in the ways we practice biomedical medicine. One example is a healthcare system’s inclusion of racial identities in clinical algorithms. Race correction is a problematic practice as it suggests race is biological rather than a social construct, and may result in either over or under treatment for individuals [25]. Additionally, race corrections can normalize poor health outcomes, which result from structural racism, of racially minoritized people. Components of the patient experience can be attributed to institutional racism, including policies and communication modalities that advantage white or other majority patients, and systematic discrimination by those who occupy institutional roles of power. For instance, increased incorporation of telemedicine into clinical care may result in a relative reduction in access to racially minoritized populations that are less likely to use these services. This part of the framework calls for interrogation of institutions that the population may enter and exit over the lifecourse, and the impact on health.

Interpersonal racism includes interactions between individuals that uphold structural racism. This form of racism involves direct acts of discrimination and bias between people, and is the most commonly studied form of racism, operationalized sometimes as major experiences, and sometimes as day-to-day experiences [26,27].

The C2DREAM framework concludes with individual-level factors: biological factors, health behaviors, and individual health. The pathway of biological factors and health behaviors impact on individual and population health is well established [26]. The compounding manifestations of the aforementioned forms of racism impact individual health, and in turn contribute to health inequities.

All forms of racism impact SDoH. Each previous level impacts subsequent levels, leading to a cumulative health effect. There are differences in severity and longevity in each form across the lifespan, frequently due to differences in space and place- geographically and/or social order [8,13]. Thus, it is necessary to consider the types of racism an individual encounters across different critical stages of their lifecourse (including across generations) and how these forms of racism may have a stronger or weaker impact upon their health. This framework goes beyond naming race as a SDoH and instead explores pathways through which racism shapes SDoH and subsequent health [1].

The C2DREAM Framework differs from existing frameworks and models, as it was designed to explicitly guide critical thinking related to CVD and its comorbidities. It considers the upstream forces of racism and how racism shapes the SDoH for multiple chronic conditions simultaneously. This framework should be used by CVD researchers, but could be adopted and applied by chronic disease researchers. Researchers should reflect on the definitions and examples provided, and then move through each part of the framework with their target population in mind, considering which concepts are most impactful and which measures can be used. Racism varies by geographical context, due to potential historical and social order differences.13 To achieve true racial health inequity requires context specific interventions. Ensuring that interventions map back to a common model can push this work forward. The C2DREAM framework combines championed theoretical approaches and provides a specific starting point for CVD researchers alike.

## Measurement

### Specifics for C2DREAM projects

A key step to understanding and eliminating the mechanisms linking racism and health is accurate measurement of racism. C2DREAM uses a combination of measures to assess the role of racism in participants' lives and communities across levels, from societal to individual/biological. Measurements fall broadly into two categories: racism measures and social, behavioral, and community measures. The appended table details measures and their designation on the conceptual pathway to multiple chronic diseases. The selected measures map onto the C2DREAM framework and operationalize key metrics related to health and SDoH. Measures are a combination of those chosen by the C2DREAM team and those chosen by a national coordinating center for all 11 NIMHD-funded centers. The correlated categories map to a form of racism.

### Racism measures

Four individual level measures operationalize *structural racism*: food insecurity, current living situation, access to health services, and health insurance coverage, as proxies for living within a society characterized by structural racism across the domains of food access, healthcare access, and housing [9]. These measures are typical in that they capture one dimension of structural racism (example- residential segregation) but not the combined impacts of the various ways structural racism shapes lives (example- how residential segregation shapes access to green spaces) [9,21]. A fifth area-level measure is the Multidimensional Measure of Structural Racism (MMSR) [28], which combines multiple unidimensional measures (residential segregation, education, employment, income, and homeownership) through latent class analysis to create a multidimensional measure of exposure to structural racism within a community. MMSR is one of the first multidimensional racism measures developed to date, and has been used to describe racial health inequities in Minnesota specifically [28].

Validated measures of institutional racism are lacking from the extant literature. These measures might tap into perceptions of policies and procedures of an organization that benefit a majority group or create barriers for people of color. Given that many chronic disease interventions focus on healthcare institutions, C2DREAM focused on healthcare institutions. Other institutional settings with important roles in health inequity include police or justice systems, workplaces, schools, and social service agencies. Lacking a validated measure of healthcare institutional level factors, we utilized an individual measure of discrimination experienced while seeking or receiving healthcare, the Discrimination in Medical Settings Scale [29]. While this measure focuses on interpersonal discrimination, it targets discrimination from medical personnel, which as a role of power, decision-making, and control of resources, represents a type of institutional racism [30]. However, institutional racism measures that target the role of policies in these and other settings are needed.

To capture exposure to interpersonal racism, C2DREAM is using the Everyday Discrimination Scale (EDS) to assess the frequency of experiences of common types of interpersonal discrimination, as well as the social identity that those experiences are attributed to.

### Social, behavioral, community, and health measures

Finally, all C2DREAM studies will collect up to three individual outcome measures. Where possible, common health problems and comorbidities are being collected by either self-report or chart review, and the PROMIS global-10 will provide an outcome measure of overall physical and mental health. Since many C2DREAM studies are focused on implementation of interventions targeting behavior change, we are also incorporating a measure of predictors of behavior change based on the Theory of Planned Behavior [31]. While items will vary study to study according to the desired behavioral outcome, the goal is to combine these data into a meaningful measure of likelihood to change behavior.

The C2DREAM framework invites researchers and practitioners within its network of organizations and studies to think more critically about the role of racism in CVD, hypertension, and obesity. The framework goes beyond naming race as a social determinant of health [1], and places exposure to racism as the underlying cause. C2DREAM acknowledges that primarily racism, but other oppressive systems as well, shape SDoH [2]. To further support this work, we must develop measures for all forms of racism, especially structural and institutional racism.

## Future directions

The goal of the C2DREAM framework is to guide researchers to critically think about and measure the role of racism across its many levels of influence to better elucidate the ways it contributes to persistent health inequities. The conceptual framework highlights the interconnectedness between forms of racism, SDoH, and the lifecourse to provide a greater context to individual health outcomes. When measures are appended to the framework (see appendix), one can notice that most measures map onto biologic/personal factors, health behaviors, and individual health outcomes, with far fewer measuring institutional or structural factors. Work is underway to expand measures in the next iteration of C2DREAM and other NIMHD-funded centers, including more focus placed on area-level measures of racism. There is a need for concise definitions and common language to describe phenomena so the field can work towards a common goal. Clarity in definitions would also lead to clarity in development of accurate measures.

The gaps in measurement highlighted here indicate where we need to focus efforts on development. Further, they indicate gaps in our knowledge that will hinder interventions; we cannot intervene upon the mechanisms linking racism and health if we have yet to identify them. Interventions will also be unsuccessful without a holistic understanding of how the different forms of racism interact. We can intervene upon physician bias through education and training, for example, but if institutional or cultural forms of racism persist, these interventions will be incomplete and likely unsuccessful as one individual will struggle against biased institutional norms.

Therefore, we need measures of structural racism that are dynamic and responsive to an individual’s unique social position across axes of race, gender, age, language, citizenship, ability, and others. These measures must capture the interactions between domains of structural racism - how does segregation impact access to health affirming resources and health care? How does policing in a neighborhood and the distrust of those institutions breed distrust in the medical system [32].

The framework outlined above illustrates key orientations to conducting anti-racist research. Whether the intention is to measure the impact of structural racism on health, or to understand how social determinants such as the built environment or access to care influence health, having a fundamental understanding of the ways in which racism, and structural racism in particular, shape the lived experiences of racialized people is essential for achieving health equity. We recognize that not all studies will measure structural racism, some will focus on institutional racism or interpersonal biases. Yet, even if racism is not a key independent variable, because US society is founded upon white supremacy and racism is ordinary and pervasive, a lack of interrogation of those forces will limit researchers’ full understanding of contributors to health inequities. To build towards this future, the framework urges researchers to think more interconnectedly and develop the needed measures to do so across multiple levels, not just the individual and interpersonal levels which are most commonly used.

Thus, reading the cited literature on structural racism, PHCRP, and the lifecourse perspective can help physicians and researchers identify structural constraints that may impede well-being. By considering how each interaction is racialized, more effective interventions can be developed. Further, if these interventions are implemented at the institutional or structural level, the effects extend far beyond any one individual. If the goal is to improve population health, then this is where these interventions must occur. Undergirding the conceptual framework, are oppressive systems of power. These power inequities bleed into all interactions, such as between a patient and physician or between a patient and the health insurance system. While intervening on these power inequities can seem daunting, interventions can begin to balance these power differentials. Physicians can truly recognize that patients are the experts of their own bodily experiences and provide patients with more agency as they discuss care options and move through the healthcare system. Affluent hospitals can accept more patients with public health insurance rather than turning them away to under-resourced hospitals, perpetuating disparities in health care quality and access.

Utilizing this framework and critically contending with the effects of racism in its multiple and cumulative forms will lead to better research and interventions. As this framework centers exposure to racism – a foundational force in the USA. Above all, we hope this framework can be used as a guide for researchers designing an intervention or study, no matter the level (structural, institutional, interpersonal, etc.), to think critically about how those participants and that study are situated within upstream, structural determinants of health.

## Supporting information

Orakwue et al. supplementary material Orakwue et al. supplementary material

## Supplementary material

The supplementary material for this article can be found at https://doi.org/10.1017/cts.2024.518.

## References

[1] Javed Z , Haisum Maqsood M , Yahya T , et al. Racism, and cardiovascular health: applying a social determinants of health framework to racial/Ethnic disparities in cardiovascular disease. Circ Cardiovasc Qual Outcomes. 2022;15(1):e007917. doi: 10.1161/CIRCOUTCOMES.121.007917. 35041484

[2] Churchwell K , Elkind MSV , Benjamin RM , et al. Call to action: structural racism as a fundamental driver of health disparities: a presidential advisory from the American heart association. Circulation. 2020;142(24):e454–e468. doi: 10.1161/CIR.0000000000000936. 33170755

[3] Breathett K , Sims M , Gross M , et al. Cardiovascular health in American Indians and Alaska natives: a scientific statement from the American heart association. Circulation. 2020;141(25):e948–e959. doi: 10.1161/CIR.0000000000000773. 32460555 PMC7351358

[4] Hardeman RR , Karbeah J. Examining racism in health services research: a disciplinary self critique. Health Serv Res. 2020;55(Suppl 2):777–780. doi: 10.1111/1475-6773.13558. 32976632 PMC7518806

[5] Alang S , Hardeman R , Karbeah J , et al. White supremacy and the core functions of public health. Am J Public Health. 2021;111(5):815–819. doi: 10.2105/AJPH.2020.306137. 33826395 PMC8033999

[6] Bailey ZD , Krieger N , Agénor M , Graves J , Linos N , Bassett MT. Structural racism and health inequities in the USA: evidence and interventions. The Lancet. 2017;389(10077):1453–1463. doi: 10.1016/S0140-6736(17)30569-X. 28402827

[7] Ford CL , Airhihenbuwa CO. Commentary: just what is critical race theory and what’s it doing in a progressive field like public health? Ethn Dis. 2018;28(Supp 1):223. doi: 10.18865/ed.28.S1.223. 30116090 PMC6092167

[8] Ford CL , Airhihenbuwa CO. Critical race theory, race equity, and public health: toward antiracism praxis. Am J Public Health. 2010;100(Suppl 1):S30–S35. doi: 10.2105/AJPH.2009.171058. 20147679 PMC2837428

[9] Groos M , Wallace M , Hardeman R , Theall KP. Measuring inequity: a systematic review of methods used to quantify structural racism. J Health Dispar Res Pract. 2018;11(2):18.

[10] Iruka IU , Gardner-Neblett N , Telfer NA , et al. Effects of racism on child development: advancing antiracist developmental science. Ann Rev Dev Psychol. 2022;4(1):109–132.

[11] CDC. Chapter 1: Models and Frameworks | Principles of Community Engagement | ATSDR. Agency for Toxic Substance and Disease Registry. Published December 6, 2018. https://www.atsdr.cdc.gov/communityengagement/pce_models.html. Accessed February 5, 2023.

[12] Alvidrez J , Castille D , Laude-Sharp M , Rosario A , Tabor D. The national institute on minority health and health disparities research framework. Am J Public Health. 2019;109(Suppl 1):S16–S20. doi: 10.2105/AJPH.2018.304883. 30699025 PMC6356129

[13] Gee GC , Walsemann KM , Brondolo E. A life course perspective on how racism may be related to health inequities. Am J Public Health. 2012;102(5):967–974. doi: 10.2105/AJPH.2012.300666. 22420802 PMC3483932

[14] Gee GC , Hing A , Mohammed S , Tabor DC , Williams DR. Racism and the life course: taking time seriously. Am J Public Health. 2019;109(S1):S43–S47. doi: 10.2105/AJPH.2018.304766. 30699016 PMC6356137

[15] Elder Jr. GH. The life course as developmental theory. Child Dev. 1998;69(1):1–12. doi: 10.1111/j.1467-8624.1998.tb06128.x. 9499552

[16] Krieger N. Methods for the scientific study of discrimination and health: an ecosocial approach. Am J Public Health. 2012;102(5):936–944. doi: 10.2105/AJPH.2011.300544. 22420803 PMC3484783

[17] Jones C. Levels of racism: a theoretic framework and a gardener’s tale. Am J Public Health. 2000;90(8):1212–1215. doi: 10.2105/AJPH.90.8.1212. 10936998 PMC1446334

[18] Lewis ME , Volpert-Esmond HI , Deen JF , Modde E , Warne D. Stress and cardiometabolic disease risk for indigenous populations throughout the lifespan. Int J Environ Res Public Health. 2021;18(4):1821. doi: 10.3390/ijerph18041821. 33668461 PMC7918141

[19] Bailey ZD , Feldman JM , Bassett MT. How structural racism works — racist policies as a root cause of U.S. racial health inequities. N Engl J Med. 2021;384(8):768–773. doi: 10.1056/NEJMms2025396. 33326717 PMC11393777

[20] Calvin R , Winters K , Wyatt SB , Williams DR , Henderson FC , Walker ER. Racism and cardiovascular disease in African Americans. Am J Med Sci. 2003;325(6):315–331. doi: 10.1097/00000441-200306000-00003. 12811228

[21] Adkins-Jackson PB , Chantarat T , Bailey ZD , Ponce NA. Measuring structural racism: a guide for epidemiologists and other health researchers. Am J Epidemiol. 2021;25(4):539–547. doi: 10.1093/aje/kwab239. PMC9077112 34564723

[22] Krieger N. Embodying inequality: a review of concepts, measures, and methods for studying health consequences of discrimination. Int J Health Serv. 1999;29(2):295–352. doi: 10.2190/M11W-VWXE-KQM9-G97Q. 10379455

[23] Bowleg L. The problem with the phrase women and minorities: intersectionality—an important theoretical framework for public health. Am J Public Health. 2012;102(7):1267–1273. doi: 10.2105/AJPH.2012.300750. 22594719 PMC3477987

[24] Dean LT , Thorpe RJ. What structural racism is (or is not) and how to measure it: clarity for public health and medical researchers. Am J Epidemiol. 2022;191(9):1521–1526. doi: 10.1093/aje/kwac112. 35792088 PMC9437815

[25] Vyas DA , James A , Kormos W , Essien UR. Revising the atherosclerotic cardiovascular disease calculator without race. Lancet Digit Health. 2022;4(1):e4–e5. doi: 10.1016/S2589-7500(21)00258-2. 34952675

[26] Peek ME. Racism and health: a call to action for health services research. Health Serv Res. 2021;56(4):569–572. doi: 10.1111/1475-6773.13693. 34155638 PMC8313951

[27] Williams DR , Lawrence JA , Davis BA. Racism and health: evidence and needed research. Ann Rev Public Health. 2019;40(1):105–125. doi: 10.1146/annurev-publhealth-040218-043750. 30601726 PMC6532402

[28] Chantarat T , Van Riper DC , Hardeman RR. The intricacy of structural racism measurement: a pilot development of a latent-class multidimensional measure. EClinicalMedicine. 2021;40:101092. doi: 10.1016/j.eclinm.2021.101092. 34746713 PMC8548924

[29] Peek ME , Nunez-Smith M , Drum M , Lewis TT. Adapting the everyday discrimination scale to medical settings: reliability and validity testing in a sample of African American patients. Ethn Dis. 2011;21(4):502–509. 22428358 PMC3350778

[30] Needham BL , Ali T , Allgood KL , Ro A , Hirschtick JL , Fleischer NL. Institutional racism and health: a framework for conceptualization, measurement, and analysis. J Racial Ethn Health Disparities. 2022;22(4):1–23. doi: 10.1007/s40615-022-01381-9. PMC9395863 35994173

[31] Ajzen I. The theory of planned behavior. Organ Behav Hum Decis Process. 1991;50(2):179–211. doi: 10.1016/0749-5978(91)90020-T.

[32] Alang S , McAlpine DD , Hardeman R. Police brutality and mistrust in medical institutions. J Racial Ethn Health Disparities. 2020;7(4):760–768. doi: 10.1007/s40615-020-00706-w. 31989532

