# Supplementary material for: The C2DREAM framework: Investigating the structural mechanisms undergirding racial health inequities
Source: J Clin Transl Sci. 2024 Apr 16;8(1):e80. doi: 10.1017/cts.2024.518 (PMC11091923; doi:10.1017/cts.2024.518)
Supplement: Orakwue et al. supplementary material [file S2059866124005181sup001.docx]

**APPENDIX**

Within each level, this framework delves into NIMDHD’s domains of influence, providing specific examples of how that form of racism may manifest in a person’s life. The dark blue boxes represent specific individual experiences. The yellow boxes represent specific occurrences in the healthcare system, which is an important site for exposure to racism in this study. The beige boxes are miscellaneous important experiences. The arrow across the bottom is intended to serve as a reminder that populations and individuals experience varying degrees and combinations of racism throughout their lifetime.

Appendix 1, Table 1. Table of Measures

| **Instrument Name** | **Framework** | **Measurement Scope** | **Type of Racism (If Applicable)** |
| --- | --- | --- | --- |
| Multidimensional Measure of Structural Racism^1^ | Structural and Social Determinants of Health | Society | Structural Racism |
| Food Insecurity (PhenX)^2, 3^ | Structural and Social Determinants of Health | Individual |  |
| Current Living Situation^4^ | Structural and Social Determinants of Health | Individual |  |
| Access Health Services (PhenX)^2, 5^ | Structural issues in healthcare | Individual |  |
| Health Insurance Coverage (PhenX)^2, 6-7^ | Structural issues in healthcare | Individual |  |
| Time since last healthcare visit^5^ | Structural Issues in Healthcare | Individual |  |
| Usual source of healthcare (Y/N)^5^ | Structural Issues in Healthcare | Individual |  |
| Usual source of healthcare (setting type)^5^ | Structural Issues in Healthcare | Individual |  |
| Emergency Department use^5^ | Structural Issues in Healthcare | Individual |  |
| Delayed medical care^5^ | Structural Issues in Healthcare | Individual |  |
| Difficulty paying for needs^4^ | Structural Issues in Healthcare | Individual |  |
| Transportation access^4^ | Structural Issues in Healthcare | Individual |  |
| Difficulty getting medications or medical supplies^4^ | Structural Issues in Healthcare | Individual |  |
| Disparate Health Care Quality (PhenX)^2, 8^ | Experience in healthcare | Individual | Institutional Racism |
| Group-Based Medical Mistrust Scale^9^ | Experience in healthcare | Community |  |
| Discrimination In Medical Settings Scale^10^ | Experience in healthcare | Individual |  |
| Brief Sense of Community Scale^11^ | Experience in community | Community | Interpersonal Racism |
| Interpersonal violence^12^ | Experience in community | Individual |  |
| Modified Buckner Neighborhood Cohesion Scale^13,14^ | Experience in community | Community |  |
| Loneliness^4^ | Experience in community | Individual |  |
| Social connectedness^4^ | Experience in community | Individual |  |
| Help with activities of daily living if needed^4^ | Experience in community | Individual |  |
| Financial abuse^4^ | Experience in community | Individual |  |
| Everyday Discrimination Scale (PhenX)^2,15^ | Experience with bias | Individual |  |
| Major Experiences Scale (PhenX)^2,16^ | Experience with bias | Individual |  |
| Lifetime Experience of Discrimination Scale^16^ | Experience with bias | Individual |  |
| Current Age (PhenX)^2,17^ | Biologic/Personal Factors | Individual | Individual Level Factors |
| Race & Ethnicity (PhenX)^2,18^ | Biologic/Personal Factors | Individual |  |
| Biological Sex (PhenX)^2,19^ | Biologic/Personal Factors | Individual |  |
| Gender Identity (PhenX)^2, 20^ | Biologic/Personal Factors | Individual |  |
| Sexual Orientation (PhenX)^2, 21^ | Biologic/Personal Factors | Individual |  |
| Education (PhenX)^2,22^ | Biologic/Personal Factors | Individual |  |
| Current Employment Status (PhenX)^2, 23^ | Biologic/Personal Factors | Individual |  |
| English Proficiency (PhenX)^2, 24^ | Biologic/Personal Factors | Individual |  |
| Health Literacy (PhenX)^2, 25^ | Biologic/Personal Factors | Individual |  |
| Income (PhenX)^2, 26^ | Biologic/Personal Factors | Individual |  |
| Birthplace (PhenX)^2, 27^ | Biologic/Personal Factors | Individual |  |
| Household size^2, 28^ | Biologic/Personal Factors | Individual |  |
| Relationship status^4^ | Biologic/Personal Factors | Individual |  |
| Disability status^29-35^ | Biologic/Personal Factors | Individual |  |
| Alcohol Use^36^ | Health Behaviors | Individual |  |
| Tobacco Use^37^ | Health Behaviors | Individual |  |
| Exercise^38, 39^ | Health Behaviors | Individual |  |
| Mental Health^40^ | Health Behaviors | Individual |  |
| Theory of Planned Behavior measure^41^ | Health Behaviors | Individual |  |
| Non-prescription drug use^42-44^ | Health Behaviors | Individual |  |
| Comorbidities and Common Medical Problems (Self-report or EHR)^45^ | Individual Health Outcomes | Individual |  |
| PROMIS Global-10^46^ | Individual Health Outcomes | Individual |  |

**References**

1. Chantarat T, Van Riper DC, Hardeman RR. The intricacy of structural racism measurement: A pilot development of a latent-class multidimensional measure. *EClinicalMedicine*. 2021;40:101092. doi:10.1016/j.eclinm.2021.101092
2. Hamilton CM, Strader LC, Pratt JG, et al. The PhenX Toolkit: get the most from your measures. Am J Epidemiol. 2011;174(3):253-260. doi:10.1093/aje/kwr193
3. Food Insecurity, <https://www.phenxtoolkit.org/protocols/view/270301?origin=search>
4. Adapted from Kaiser Permanente Your Current Life Situation (KP YCLS) (shorter form) Q1)
5. Access to Health Services, <https://www.phenxtoolkit.org/protocols/view/270101?origin=search>
6. Health Insurance Coverage, <https://www.phenxtoolkit.org/protocols/view/11502?origin=search>
7. Health Reform Monitoring Survey (HRMS), Quarter 1 2015 Questionnaire, Question numbers Q7, Q8, Q8b. <http://hrms.urban.org/survey-instrument/HRMS-Quarter-1-2015-survey.pdf>
8. Disparate Health Care Quality, <https://www.phenxtoolkit.org/protocols/view/280101?origin=search>
9. Thompson HS, Valdimarsdottir HB, Winkel G, Jandorf L, Redd W. Group-Based Medical Mistrust Scale. Published online 2004. doi:10.1037/t16933-000
10. Peek ME. Racism and health: A call to action for health services research. Health Serv Res. 2021;56(4):569-572. doi:10.1111/1475-6773.13693
11. Peterson NA, Speer PW, McMillan DW. Brief Sense of Community Scale. Published online 2008. doi:10.1037/t36645-000
12. (Adapted from Kaiser Permanente Your Current Life Situation (KP YCLS) (shorter form) Add'l Q9)
13. LaForge K, Gold R, Cottrell E, et al. How 6 Organizations Developed Tools and Processes for Social Determinants of Health Screening in Primary Care: An Overview. J Ambul Care Manage. 2018;41(1):2-14. doi:10.1097/JAC.0000000000000221
14. Buckner, JC. "The development of an instrument to measure neighborhood cohesion." American journal of community psychology 16.6 (1988): 771-791.
15. Williams, D.R., Yu, Y., Jackson, J.S., and Anderson, N.B. “Racial Differences in Physical and Mental Health: Socioeconomic Status, Stress, and Discrimination.” Journal of Health Psychology. 1997; 2(3):335-351.
16. Williams, D.R., González, H.M., Williams, S., Mohammed, S.A., Moomal, H, Stein, D.J. “Perceived Discrimination, Race and Health in South Africa: Findings from the South Africa Stress and Health Study.” Social Science and Medicine, 2008; 67: 441-452.
17. Current Age, <https://www.phenxtoolkit.org/protocols/view/10101?origin=search>
18. Ethnicity and Race, <https://www.phenxtoolkit.org/protocols/view/11901?origin=search>
19. Sex Assigned at Birth, <https://www.phenxtoolkit.org/protocols/view/11601?origin=search>
20. Gender Identity, <https://www.phenxtoolkit.org/protocols/view/11801?origin=search>
21. Sexual Orientation, <https://www.phenxtoolkit.org/protocols/view/11701?origin=search>
22. Educational Attainment, <https://www.phenxtoolkit.org/protocols/view/11002?origin=search>
23. Current Employment Status, <https://www.phenxtoolkit.org/protocols/view/11301?origin=search>
24. English Proficiency, <https://www.phenxtoolkit.org/protocols/view/270201?origin=search>
25. Health Literacy, <https://www.phenxtoolkit.org/protocols/view/270401?origin=search>
26. Annual Family Income, <https://www.phenxtoolkit.org/protocols/view/11102?origin=search>
27. Birthplace, <https://www.phenxtoolkit.org/protocols/view/10201?origin=search>
28. Adapted from PhenX - Annual Family Income [PX011102]
29. (Adapted from https://www.cdc.gov/brfss/questionnaires/pdf-ques/2021-BRFSS-Questionnaire-1-19-2022-508.pdf - Core Section 10: Disability Q#: CDIS.01)"
30. (Adapted from https://www.cdc.gov/brfss/questionnaires/pdf-ques/2021-BRFSS-Questionnaire-1-19-2022-508.pdf - Core Section 10: Disability Q#: CDIS.02)"
31. (Adapted from https://www.cdc.gov/brfss/questionnaires/pdf-ques/2021-BRFSS-Questionnaire-1-19-2022-508.pdf - Core Section 10: Disability Q#: CDIS.04)"
32. (Adapted from https://www.cdc.gov/brfss/questionnaires/pdf-ques/2021-BRFSS-Questionnaire-1-19-2022-508.pdf - Core Section 10: Disability Q#: CDIS.05)"
33. (Adapted from Item: COM_SS in The Washington Group Short Set on Functioning (WG-SS))"
34. (Adapted from https://www.cdc.gov/brfss/questionnaires/pdf-ques/2021-BRFSS-Questionnaire-1-19-2022-508.pdf - Core Section 10: Disability Q#: CDIS.03; CMS AHS HRSN Item 25; LOINC 69858-9)"
35. (Adapted from https://www.cdc.gov/brfss/questionnaires/pdf-ques/2021-BRFSS-Questionnaire-1-19-2022-508.pdf - Core Section 10: Disability Q#: CDIS.06; CMS AHS HRSN Item 26; LOINC 69861-3)"
36. (Adapted from CMS AHS HRSN Item 19; LOINC 68517-2)
37. (Adapted from CMS AHS HRSN Item #20; LOINC: 96842-0; PATH]
38. (Adapted from CMS AHS HRSN Item 17; LOINC: 89555-7)
39. (Adapted from CMS AHS HRSN Item 18; LOINC: 68516-4)
40. (Adapted from CMS AHS HRSN Item 23B; LOINC 44255-8)
41. Ajzen I. The theory of planned behavior. Organizational Behavior and Human Decision Processes. 1991;50(2):179-211. doi:10.1016/0749-5978(91)90020-T
42. Adapted from CMS AHS HRSN Item 21; LOINC 95530-2)
43. (Adapted from CMS AHS HRSN Item 22; LOINC 68524-8)
44. (Adapted from https://www.cdc.gov/brfss/questionnaires/pdf-ques/2021-brfss-questionnaire-1-19-2022-508.pdf)
45. Charlson ME, Pompei P, Ales KL, MacKenzie CR. A new method of classifying prognostic comorbidity in longitudinal studies: development and validation. J Chronic Dis. 1987;40(5):373-383. doi:10.1016/0021-9681(87)90171-8
46. Hays RD, Bjorner JB, Revicki DA, Spritzer KL, Cella D. Development of physical and mental health summary scores from the patient-reported outcomes measurement information system (PROMIS) global items. Qual Life Res. 2009;18(7):873-880. doi:10.1007/s11136-009-9496-9
